# The Medical Society

**Published:** 1897-02-27

**Authors:** 


### THE MEDICAL SOCIETY.

Professor Victor Horsley read a paper last Monday on "Traumatic Neurasthenia."

It was important to bear in mind that the symptoms of this condition were those which occurred in ordinary or idiopathic neurasthenia. They might come on immediately after the accident which gave rise to them, or they might not occur till seven or fourteen or more days had elapsed. In the acute cases which came on directly after the injury the symptoms might at first be mixed with those due to concussion, and some of them might be the result of a direct lesion of the brain.

A most remarkable symptom of this acute form of neurasthenia was the variation which occurred in the temperature. A condition was apt to arise which was precisely analogous to the neurotic pyrexia described by various authors. The temperature would suddenly shoot up to a great height, 110° Fahr. and 111° Fahr., and at the acme of the pyrexia there might be dulness of intellect, hallucinations, and sensations of great heat. It was to be noted, however, that in these cases the temperature was rapidly brought down by the application of cold, which was not the case with the pyrexia due to organic mischief resulting from traumatism.

The latent period which elapsed before the symptoms developed was a most important and characteristic symptom in some cases. For two or three days after the injury patients would declare that they were quite well, and only after some time would incapacitating symptoms develop. These symptoms, whether taking the form of local paralysis, or of paralysis combined with over-action, were strictly analogous to those of idiopathic neurasthenia or grave hysteria.

In many cases which had been honestly enough attributed in the witness-box to organic injury the pain would be found to correspond accurately to the hysterogenetic zones described by Charcot.

Knee jerks, clonus, the micturition, and even the percentage composition of the urine might all be affected, and yet the case might be one of purely functional disorder of the nervous system. Subjective sensation of pain, especially pain in the dorso-lumbar region, aggravated by jar, often led not only to a diagnosis of local injury, but, what was far more important, to treatment by counter irritation and by spinal supports, both of which were to be deprecated. The one treatment which was of real use was that by isolation, massage, and over-feeding, which was associated with the name of Weir Mitchell.

In considering the prognosis of these cases, it must always be borne in mind that the statement so often made in the witness-box that they invariably recovered was not true. They did not always recover, and when they did they often relapsed, small causes of nervous disturbance bringing up again the old troublesome train of symptoms.

